# Case Report: Orbital and intracranial inflammatory syndrome in rheumatoid arthritis

**DOI:** 10.3389/fopht.2026.1929961

**Published:** 2026-09-29

**Authors:** Cecelia Mans, Safa Ibrahim, Dina Abdelsalam, Andrew G. Lee

**Affiliations:** 1 College of Osteopathic Medicine, Western University of Health Sciences, Lebanon, OR, United States; 2 Department of Ophthalmology, Blanton Eye Institute, Houston Methodist Hospital, Houston, TX, United States; 3 Department of Ophthalmology, Cullen Eye Institute, Baylor College of Medicine, Houston, TX, United States; 4 Departments of Ophthalmology, Neurology, and Neurosurgery, Weill Cornell Medicine, New York, NY, United States; 5 Department of Ophthalmology, University of Texas MD Anderson Cancer Center, Houston, TX, United States; 6 Texas A&M College of Medicine, Bryan, TX, United States; 7 Department of Ophthalmology, University of Iowa Hospitals and Clinics., Iowa City, IA, United States

**Keywords:** cavernous sinus, inflammatory orbital disease, rheumatoid arthritis, rheumatoid vasculitis, steroid-dependent inflammation

## Abstract

**Purpose:**

To describe a case of rheumatoid arthritis (RA)-associated orbital and intracranial inflammatory disorder (OIID).

**Background:**

RA is a systemic autoimmune disease associated with a multitude of extra-articular manifestations. Cigarette smoking increases the risk of RA vasculitis and adversely influences the expression of the proto-oncogene p53 in RA. Although ocular manifestations of RA are well known, orbital and intracranial inflammatory presentations are much less common.

**Case presentation:**

A 63-year-old woman with seropositive RA and a significant tobacco smoking history presented with intermittent jaw pain, headache, left temporal tenderness, blurred vision of the left eye, and intermittent diplopia. Serum laboratory studies demonstrated elevated RF and erythrocyte sedimentation rate (ESR). Cranial and orbital magnetic resonance imaging (MRI) showed left orbital apex enhancement with extension into the left cavernous sinus and Meckel cave. The patient improved with intravenous methylprednisolone and high-dose prednisone, but symptoms recurred with each steroid taper. 13 months later, the patient developed new contralateral right-sided orbital inflammation with cavernous sinus and superior orbital fissure involvement.

**Conclusions:**

Clinicians should be aware that orbital (e.g., orbital inflammatory pseudotumor) and intracranial presentations may occur in RA in addition to the more common ocular (e.g., scleritis) presentations.

## Introduction

Rheumatoid arthritis (RA) is a systemic, bilateral, progressive autoimmune disease that can be associated with extra-articular manifestations (1). The precise pathophysiology of these extra-articular presentations remains ill-defined and is likely multifactorial including environmental, genetic, and immune factors (2). Inflammatory ocular disease in RA is well described and can affect the episclera, sclera, cornea, uvea, and retina (3). Orbital presentations of RA, however, are uncommon, and intracranial involvement is rare (3). Management of inflammatory ocular disease secondary to RA is generally managed by disease-modifying antirheumatic drugs (e.g., alkylating agents and high-dose glucocorticoids) (3).

## Case presentation

A 63-year-old white woman with seropositive RA presented with intermittent jaw pain, headache, tenderness over the left temporal area, blurred vision of the left eye (OS), and intermittent binocular diplopia. Past medical history included atrial arrhythmia, breast cancer, and osteoporosis. Social history included a one-pack-per-day smoking habit. Her medications included alendronate, folic acid, furosemide, infliximab, methotrexate, sertraline, sulfamethoxazole–trimethoprim, and Xarelto.

On examination, her visual acuity measured 20/40 in the right eye (OD) and 20/30 OS. Humphrey visual field testing revealed a mean deviation of −0.50 decibels (dB) in the right eye, and −3.03 dB in the left eye with a corresponding inferior arcuate defect. The pupil exam showed a relative afferent pupillary defect OS. Slit lamp biomicroscopy showed no uveitis. Ocular motility exam showed a −2 limitation of adduction and abduction OS, but the right eye moved normally. Ophthalmoscopy was normal in both eyes (OU). The remainder of the eye exam was normal OU. Optical coherence tomography (OCT) showed retinal nerve fiber layer (RNFL) measurements of 91 microns OD and 96 microns OS.

Cranial and orbital magnetic resonance imaging (MRI) with contrast showed asymmetric enhancement of the left orbital apex involving the left superior orbital fissure with extension to the cavernous sinus and right Meckel cave (**Figure 1A**). A temporal artery biopsy showed a negative result for giant cell arteritis. Intravenous high-dose methylprednisolone followed by high-dose prednisone with a slow taper was initiated. At follow-up 3 months later, repeat cranial and orbital MRI showed near resolution of left orbital apex enhancement but persistent enhancement at the right Meckel cave (**Figure 2**). The patient was started on maintenance infliximab infusions.

**Figure 1:**
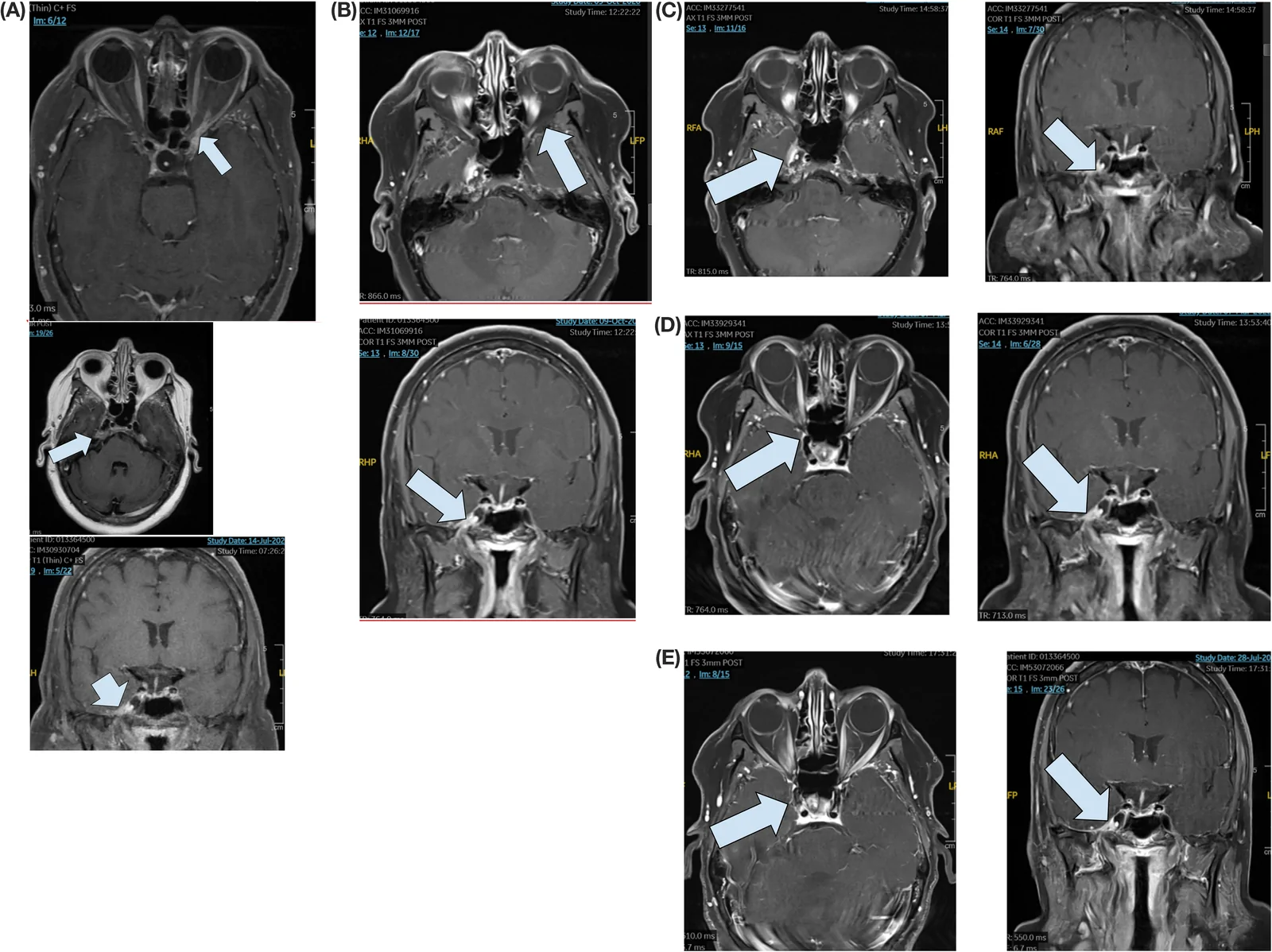
Serial post-contrast T1-weighted, fat-suppressed MRI of the orbits and brain. Arrows indicate the predominant site of abnormal enhancement at each time point. Note that abnormal enhancement is centered on the orbital apex, superior orbital fissure, cavernous sinus, and Meckel cave (i.e., the orbital apex–skull base–cavernous sinus junction) rather than the anterior/mid intraorbital compartment. All panels are post-contrast, fat-suppressed T1-weighted images. Acquisition protocols differed across time points (thin-section vs. 3-mm sections; varying TRs; axial and coronal planes). The most comparable available sections were chosen for display. Apparent interval changes should be interpreted considering these technical differences. **(A)** Initial presentation: asymmetric enhancement of the left orbital apex and superior orbital fissure with extension to the left cavernous sinus and right Meckel cave (arrow, cavernous sinus/Meckel cave). **(B)** Three months later: near-complete resolution of the left orbital apex enhancement; unchanged enhancement at the anterior/inferior right Meckel cave. **(C)** Thirteen months: increased enhancement of the right cavernous sinus, foramen ovale, and superior orbital fissure, with decreased enhancement at the right orbital apex, orbit, and lateral rectus (arrow, cavernous sinus). **(D)** Twenty months: stable asymmetric enhancement of the right cavernous sinus with mild periventricular white-matter signal change. **(E)** Five years: mild, grossly stable enhancement of the right cavernous sinus inferolaterally.

**Figure 2:**
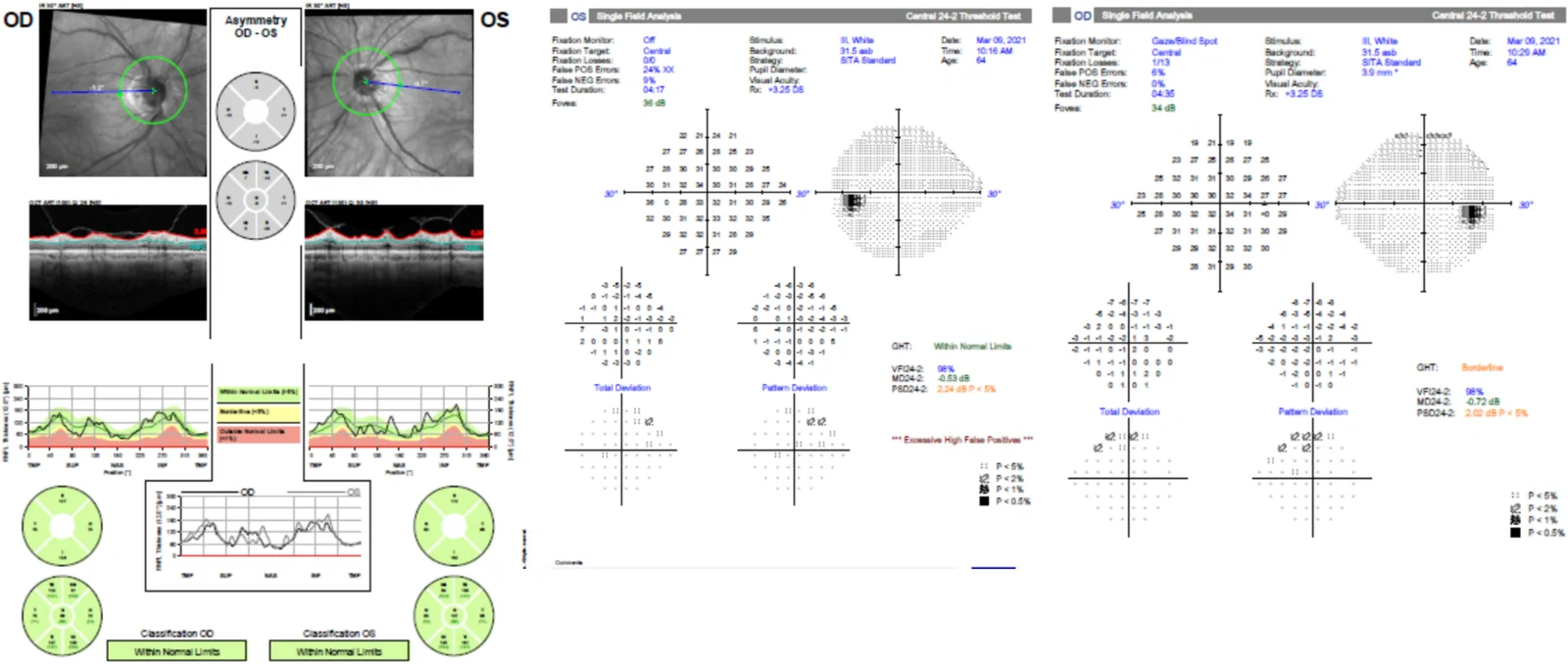
OCT and Humphrey visual field 3 months after initial presentation.

13 months after the initial presentation, the patient developed right-eye chemosis, pain, ophthalmoplegia, ptosis, and lid edema. At this time, her visual acuity was 20/20 OU without RAPD or anisocoria. Repeat orbital and cranial MRI revealed increased enhancement of the right orbital apex, cavernous sinus, foramen ovale, and superior orbital fissure (**Figure 1B**). High-dose steroid treatment was reinitiated.

Testing for inflammatory and infectious etiologies showed a negative finding, including angiotensin-converting enzyme (ACE), lysozyme, myelin oligodendrocyte glycoprotein, neuromyelitis optica (NMO), tuberculosis, Lyme disease, and immunoglobulin (IgG4). Lumbar puncture showed normal cerebrospinal fluid. The serum rheumatoid factor (RF) was elevated at 88, and the C-reactive protein (CRP) level was elevated at 1.05. Orbital MRI still showed stable enhancement of the right orbit and cavernous sinus (**Figure 1C**). She again improved with IV methylprednisolone and high-dose prednisone but experienced recurrent diplopia with each steroid taper in the following months, notably at about 10 mg of prednisone. At the last follow-up, the patient was stable on leflunomide and hydroxychloroquine with stable orbital and cranial imaging and stable visual function OU (**Figures 1D**, **E**, **3**).

**Figure 3:**
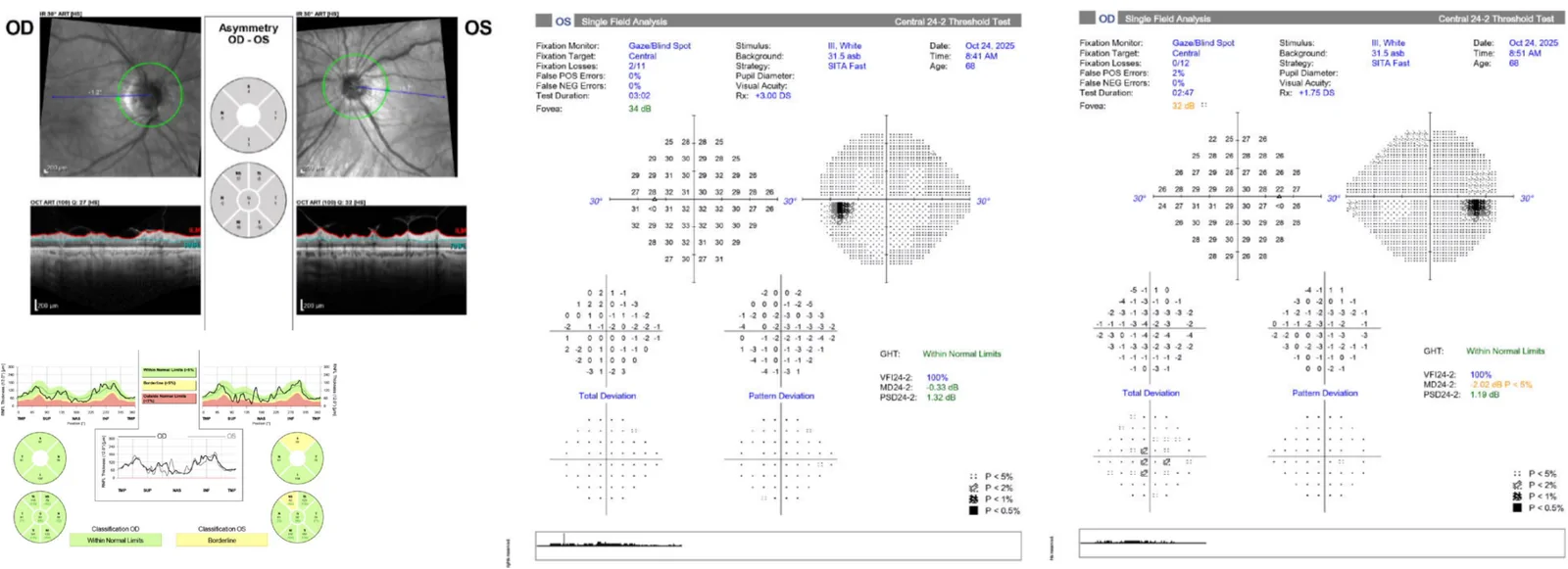
OCT and Humphrey visual field 5 years after initial presentation.

## Discussion

RA-associated orbital inflammatory syndrome is a diagnosis of exclusion. Any lesion producing painful ophthalmoplegia in the orbital apex, superior orbital fissure, or cavernous sinus or with Meckel cave involvement requires workup across the following etiologic categories: inflammatory, neoplastic, infectious, and vascular. Differentials for this case include IgG4-related disease, sarcoidosis, granulomatosis with polyangiitis (GPA), lymphoma, and Tolosa–Hunt syndrome (THS). IgG4-related disease can account for bilateral orbital inflammation. This case revealed negative serum IgG4 and normal lumbar puncture. There was no lacrimal/infraorbital nerve or systemic involvement described, although the most definitive exclusion would require a biopsy that was not performed. Sarcoidosis was unlikely because serum ACE and lysozyme markers showed negative results, CSF showed a normal finding, and there were no pulmonary or hilar findings. GPA can occur as an orbital mass; however, our patient did not have sinonasal or upper-airway disease, bone destruction, or ANCA-positive markers. The multiyear relative stability and steroid dependence argue against a lymphoma diagnosis. THS is idiopathic, and the presence of seropositive RA reclassifies this case as a secondary inflammatory syndrome rather than THS.

Initial workup for orbital and cavernous sinus inflammation includes MRI of orbits and brain without and with contrast. Dedicated views for intracranial extension can be added. Relevant for GPA, CT can complement MRI for bone erosion. Diffusion-weighted imaging is useful when lymphoma is considered. Serology lab includes CBC, CMP, ESR, CRP, RF/anti-CCP, ANCA, ACE, lysozyme, serum IgG4 and IgG subclasses, TSH/thyroid antibodies, ACh-receptor antibodies, and infectious studies for tuberculosis, syphilis, and Lyme. CSF analysis can exclude neoplastic and infectious meningeal processes. Biopsy can be performed in a standard workup because MRI is non-specific and radiographic persistence or steroid failure raises concern for neoplasm. Tissue diagnosis should be strongly considered in atypical, refractory, or radiographically persistent cases, as seen in our patient.

First-line treatment for orbital inflammatory syndrome includes high-dose corticosteroids like IV methylprednisolone followed by an oral prednisone taper. Treatment can provide rapid symptomatic improvement, and relapse and steroid dependence are common (4). Rituximab has the strongest evidence in refractory orbital inflammation (5). In a tertiary cohort of orbital inflammatory disease, corticosteroids achieved full remission in 50% of patients, DMARDs in a minority for myositis, and rituximab full remission in over 80% of patients (6). Specific patient populations that are at a higher risk for vasculitis secondary to rheumatoid arthritis include those with seropositivity, like a high-titer RF or anti-CCP, long-standing, erosive severe disease with nodules or prior DMARD exposure, and male sex (7). Extra-articular RA is tied to more active, severe disease; therefore, tighter control of systemic inflammation and earlier DMARD therapy can reduce the incidence of vasculitis and other extra-articular features over time (8, 13). Only one prospective interventional trial in orbital inflammatory disease is the phase 1/2 rituximab RCT (NCT00415506) (5). Epidemiologic data needed to power future trials include validated orbital disease grading and corticosteroid-sparing endpoints to enable multicenter enrollment of individually rare cases.

Limitations of this case are that a biopsy was not performed due to an unfeasible biopsy location, and this case remains a presumptive diagnosis of RA-associated orbital inflammatory syndrome. Serial MRI was acquired using non-identical protocols across time points, limiting direct quantitative longitudinal comparisons. Standardized orbital MRI protocols would strengthen future longitudinal assessments. Orbital and intracranial inflammatory syndromes associated with RA are rare, and only a limited number of cases have been reported in the literature. The list of prior cases (**Table 1**) in the literature shows similar clinical features to the current case. In the previously reported cases, patients presented with orbital symptoms of ptosis, proptosis, diplopia, pain, foreign-body sensation, and restricted extraocular movements. These symptoms parallel the initial and recurrent manifestations of orbital apex-cavernous sinus inflammation observed in our patient. The majority of reported cases occurred in adults. One pediatric pseudotumor case associated with juvenile RA has been described.

**Table 1:** Previous cases of inflammatory orbital disease associated with rheumatoid arthritis.

Article	Diagnosis	Presenting symptom	Intervention	Outcome
Sorab et al. (9)	Migratory orbital inflammation secondary to RA	Foreign-body sensation OS	1 g IVMP × 2 days. Initiated rituximab and oral steroid taper	Monitored for 1+ year without recurrence and placed on adalimumab
Nakamura et al. (10)	Orbital inflammatory pseudotumor secondary to RA	High serum RF, OD daily swelling, right proptosis, ptosis, restricted eye movement, MRI inflammation in right orbit	30 mg/day prednisolone	Gradual improvement
Ibrahim et al. (11)	IOI secondary to RA	4-month history of diplopia, right-side proptosis and ptosis, a restriction of movement in the right eye	IVMP 250 mg × 3. MTX 5 mg/week. Folic acid 5 mg 3 days/week. Rituximab infusions (1000 mg × 2) administered 2 weeks apart	A couple of months later vision had improved and ptosis resolved
Mahdaviani et al. (12)	Orbital pseudotumor secondary to RA	Fevers, arthritis, parvoviral infection, lytic bone lesions, bilateral anterior uveitis, band keratopathy, migratory polyarthritis	NA	NA
Panfilio et al. (15)	Orbital myositis in RF-positive patient	1-month of headache with gradual diplopia, paralysis of the left rectus medialis muscle and right rectus medialis muscle	Methylprednisolone 80mg daily for 2 months, returned with bilateral ophthalmoplegia and normal MRI, restarted methylprednisolone 80mg daily	Paretic rectus lateralis muscle one year after symptoms begun

Glucocorticoids, intravenous methylprednisolone, or oral prednisolone was the primary initial therapy in most prior reports and led to partial or complete short-term improvement in the prior cases. Cases reported by Nakamura et al. (10) and Ibrahim et al. (11) demonstrated that symptoms gradually improved with resolution following steroid therapy. Nakamura et al. (10) did not require a combination of disease-modifying antirheumatic drugs or biological agents, such as methotrexate or rituximab, whereas the resolution of Ibrahim et al.’s (11) case did. Our case also did not model the same treatment progression and resolution with glucocorticoids and immunosuppressive therapy. Rather, our patient experienced a steroid-dependent disease course with recurrent orbital and intracranial inflammation occurring during prednisone tapering despite additional escalation of immunosuppressive therapy. Unlike most prior cases that were unilateral and confined to the orbit, our case demonstrated bilateral sequential involvement with extension into intracranial structures, including the cavernous sinus, superior orbital fissure, and Meckel’s cave.

Prior reports generally described resolution or sustained remission after treatment, with Sorab et al.’s (9) case demonstrating remission for over 1 year. Our patient required immunomodulatory therapy consisting of leflunomide and hydroxychloroquine to maintain disease stability. Our case shows variability of RA-associated orbital inflammation when comparing prior reported cases. Although many cases respond favorably to corticosteroids, a subset may follow a chronic, steroid-dependent, relapsing course with the possibility of intracranial extension (14). Recognition of this potential disease trajectory is important for prognosis, long-term management, and monitoring. This case exemplifies the importance of counseling patients with RA about potential inflammatory manifestations and the progressive, unrelenting nature of their disease beyond initial presenting symptoms.

## Data Availability

The original contributions presented in the study are included in the article/supplementary material. Further inquiries can be directed to the corresponding author.
